# Antibody conjugates in neuroblastoma: a step forward in precision medicine

**DOI:** 10.3389/fonc.2025.1548524

**Published:** 2025-03-10

**Authors:** Jihane Balla, Carlotta Siddi, Maria Scherma, Paola Fadda, Simona Dedoni

**Affiliations:** ^1^ Department of Biomedical Sciences, Division of Neuroscience and Clinical Pharmacology, University of Cagliari, Cagliari, Italy; ^2^ Neuroscience Institute, National Research Council of Italy (CNR), Cagliari, Italy

**Keywords:** neuroblastoma, antibody-drug conjugates, antibody-fluorophore conjugates, immunotoxin, precision medicine, radioimmunotherapy

## Abstract

Neuroblastoma (NB) is a pediatric cancer that often manifests in a high-risk form and is characterized by frequent relapses and resistance to conventional therapies. This underscores the urgent need for more effective and targeted treatment strategies. One promising avenue has been the identification of unique or overexpressed surface antigens on neoplastic cells, which has facilitated the development of antibody conjugates and related technologies. These include antibody-drug conjugates (ADCs) and immunotoxins (ITs), which deliver cytotoxic agents directly to tumor cells, as well as antibody-fluorophore conjugates (AFCs), which bind to surface antigens with high specificity to target malignant tumors. Additionally, radioimmunotherapy (RIT) allows the precise delivery of radioactive isotopes linked to a monoclonal antibody directly to the tumor cells. ADCs, ITs, and RIT represent a novel class of anti-cancer agents offering precision therapy with reduced systemic toxicity, enabling longer and potentially more effective treatment regimens. Meanwhile, AFCs are valuable tools in diagnostics, aiding in detecting and characterizing malignant tissues. Despite advancements in antibody conjugates for NB, significant challenges persist, including optimizing payload delivery, mitigating off-target effects, and addressing tumor heterogeneity. Future research should also prioritize refining and integrating these technologies into multimodal treatment protocols to improve outcomes for pediatric NB patients.

## 1 Introduction

**Figure 1:**
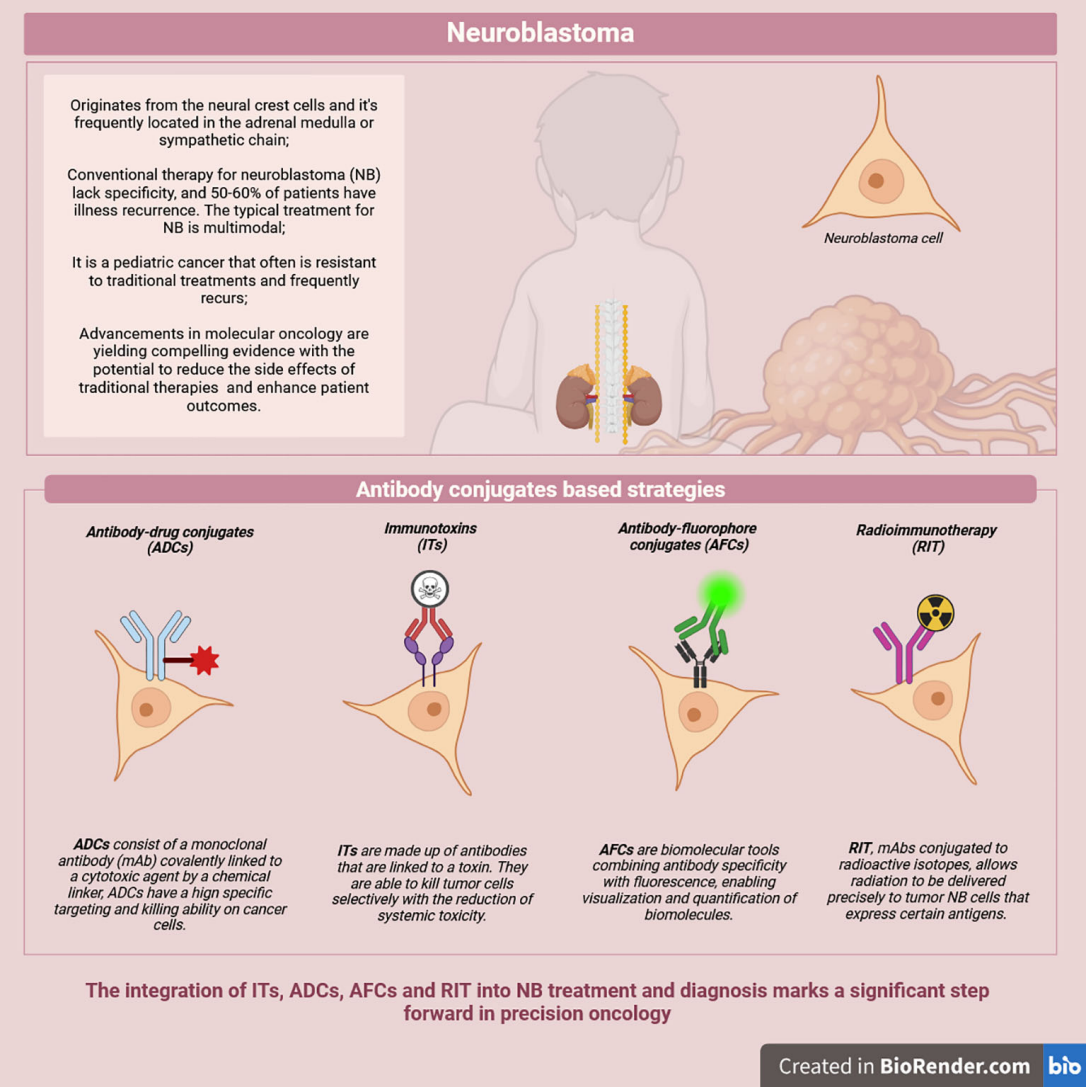
Overview of possible antibody-conjugates based strategies.

Neuroblastoma (NB) is a highly aggressive pediatric tumor that arises from neural crest cells of the sympathetic nervous system, accounting for approximately 8-10% of juvenile cancers and 15% of pediatric fatalities (1). Around the ages of 17 and 18 months, NB is frequently identified; it rises during the first year following birth and subsequently declines with age. Nearly 90% of all cases involve children younger than five (1). While it is very low in South and East Asia, the prevalence is higher in high-income nations and among certain ethnic groups. The frequency is 11.5 per million for white children and 8.5 per million for black children in the United States. Unfortunately, the prevalence and mortality rates of NB have not yet been the subject of a systematic global assessment (1). The prevalence and mortality rates among children with NB aged 0-14 years were analyzed by the Global Burden of Disease database. A total of 5560 cases and 1977 fatalities were reported in 2021. Incidence increased by 30.26%, and mortality by 20.35% from 1990 to 2021 (1). Its clinical behavior varies from spontaneous regression to relentless progression, influenced by tumor genetics, patient age, and disease stage. High-risk NB (HR-NB), often metastatic at diagnosis, has a poor prognosis, with a 5-year survival rate of 40–50% (2). Relapsed or therapy-resistant HR-NB survival drops to ~20%, highlighting the urgent need for better treatments (2). The standard treatment for HR-NB relies on multimodal protocols, involving intensive chemotherapy, surgical resection, radiation therapy, and hematopoietic stem cell transplantation, alongside targeted therapies addressing MYCN amplification, anaplastic lymphoma kinase (ALK) mutations, and signaling pathway abnormalities (3, 4). Epigenetic modulators and therapies targeting norepinephrine and somatostatin receptors are also under investigation for their potential to improve outcomes (5, 6). Despite these advances, 50-60% HR-NB patients face recurrence with limited curative options for relapsed or resistant cases. Conventional therapies lack specificity, indiscriminately targeting dividing cells, causing severe side effects and fostering chemoresistance (CR) (7). These challenges underscore the need for novel therapeutic approaches that combine efficacy with precision to minimize collateral damage and improve patient outcomes. Advances in molecular oncology provided valuable insights into potential solutions to address these limitations. One promising avenue is the identification of tumor-specific or overexpressed surface antigens on NB cells (6). These discoveries have facilitated the development of targeted therapies and diagnostic tools such as antibody conjugates (ACs), including antibody-drug conjugates (ADCs), immunotoxins (ITs), antibody-fluorophore conjugates (AFCs) and Radioimmunotherapy (RIT) (8–12) (**Figure 1**). By combining these innovative approaches with traditional therapies, there is potential to enhance therapeutic efficacy, address CR, and minimize the side effects of standard treatments. These emerging strategies provide renewed hope for improving outcomes in HR-NB. This review focuses on the latest progress made regarding the use of ACs for NB, underscoring their transformative potential in enhancing precision therapy and diagnosis.

## 2 Antibody conjugates in NB

ACs are advanced biopharmaceutical tools designed for targeted cancer diagnosis and therapy. Conjugating antibodies to a toxin, a cytotoxic drug, a radioactive isotope, or labeling molecules such as fluorescent dyes allow specific binding to targets exclusively expressed on tumorous cells for diagnosis, monitoring, or treatment (8–12) (**Figure 1**).

### 2.1 Antibody-drug conjugates in NB

#### 2.1.1 Latest insights in ADCs use for NB

While the use of monoclonal antibodies (mAb) revolutionized cancer therapy, they are often unable to reach the same level of efficacy displayed by classical treatments such as chemotherapy (13–15). This was successfully addressed by the development of ADCs. These drug-delivery systems are comprised of a mAb, a chemical linker, and a cytotoxic compound referred to as the payload. ADCs composition allows it to combine both the specificity of mAb and the potent cytotoxicity of the conjugated payload to meticulously achieve precise tumor eradication, avoiding both adverse effects and treatment resistance typically seen in classical cancer therapies (15, 16). ADCs gained clinical relevance with FDA approval of Gemtuzumab ozogamicin (Mylotarg^®^) in 2000. Since then, 14 more ADCs have been approved, underscoring their growing role in precision cancer treatment (15). Understanding NB’s complex biology is vital for precision medicine. Targeting key genetic alterations like ALK mutations and MYCN amplification with ADCs can improve outcomes. MYCN, a member of the MYC oncogene family, drives NB tumorigenesis by forming a transcriptionally active heterodimer with MAX, making it a therapeutic target (17–19). Bibbo et al. advanced Omomyc, a protein disrupting MYC-MAX interaction, by conjugating it to a HER3-targeting mAb (EV20) (20–22). This ADC, using a bifunctional linker, ensured Omomyc delivery only inside tumor cells, blocking MYC-MAX formation (22). In a pseudo-metastatic NB mouse model, EV20/Omomyc significantly reduced liver metastases, supporting its efficacy in managing NB metastasis and pediatric cancers (22) (**Table 1**). On the other hand, the development of an ADC targeting the ALK receptor offers significant advantages over traditional treatments for NB. ALK belongs to the insulin tyrosine kinase family and promotes mitogenic and anti-apoptotic effects by activating several downstream signaling pathways. These include phospholipase C-γ (PLCγ), janus kinase 3 (JAK3)-STAT3, extracellular signal-regulated kinase 1/2 (ERK1/2), and phosphatidylinositol 3-kinase (PI3K)-Akt. Mutations in ALK, such as F1174L and R1275Q, lead to constitutive activation of pro-survival pathways or receptor amplification, driving sustained tumor growth, cell proliferation, and migration. This associates the receptor with poor prognosis, making it a critical target for therapeutic intervention (23, 24). Research data shows that the ADC, CDX-0125-thienoindole (TEI), exhibited efficient antigen binding and internalization and demonstrated cytotoxicity in various NB cell lines (25). *In vivo*, CDX-0125-TEI significantly reduced tumor growth in NB mouse models, effectively targeting both wild-type (WT) and mutant ALK in patient-derived xenografts while sparing healthy cells due to its specificity (25). This dual efficacy against WT and mutant ALK broadens its applicability (**Table 1**). Besides focusing on key genetic aberrations in NB, precise therapy also leverages the targeting of surface proteins that are either overexpressed or uniquely expressed in cancer cells. One such target is B7-H3, a type I transmembrane glycoprotein linked to metastasis and poor outcomes in several cancers, including NB. Its ability to suppress T-cell activation leads to the successful immune evasion of the tumor while its contribution to the prompting of pathways such as the PI3K/Akt and STAT3 pathways can enhance survival and proliferation, further increasing the tumor’s resistance (26, 27). The ADC m276-SL-PBD, targeting B7-H3, extended event-free survival (EFS), reduced tumor volume in NB xenograft models, and showed low toxicity, confirming its tolerability and potency (28) (**Table 1**). Vobramitamab duocarmazine (vobra duo) is an anti-B7-H3 ADC showing preclinical efficacy in various solid cancers (29) and is under clinical evaluation for advanced castration-resistant metastatic prostate cancer (30). It induces apoptosis in multiple human NB cell lines (31). In co-culture experiments, B7-H3-unreactive murine NB NX-S2-luc cells showed reduced survival due to the ADC’s strong bystander activity. Using orthotopic, pseudo-metastatic, and tumor-resected NB mouse models, vobra duo delayed tumor growth and improved survival (31) (**Table 1**). Compared to Topotecan-Temozolomide (TOTEM), vobra duo delayed tumor relapse more effectively. Combined with TOTEM, it further slowed tumor progression and increased survival, enhancing conventional therapy (31). Repeated treatments in relapsed NB mice demonstrated superior efficacy and reduced off-target effects compared to TOTEM (31). Another ADC targeting a surface receptor, the Leucine-rich repeat-containing G-protein-coupled receptor 5 (LGR5), was assessed for its anti-NB action both *in vitro* and *in vivo* (32). LGR5 is a stem cell marker that is found to be overexpressed in various malignancies (33, 91). It enhances tumor cell survival, migration, and drug resistance in NB by potentiating the WNT signaling pathway (34). LGR5 was identified as a target for the treatment of NB thanks to its high expression in NB cell lines, as well as in primary tumor samples from NB patients (32). This has inspired the development of the anti-LGR5 ADC conjugated to pyrrolobenzodiazepine (PBD), a DNA alkylator. Assessment of the latter *in vitro* and *in vivo* using clinically relevant doses in mouse models xenografted with LGR5-positive human NB cell lines and PDX has led to the inhibition of tumor growth. This was attributed to a reduction in proliferation as well as an increase in apoptosis (32). Glypican 2 (GPC2), an MYCN-regulated oncoprotein overexpressed in NB cells, is associated with reduced overall survival and event-free survival (35, 36). In NB-PDX mouse models treated with a GPC2-targeting ADC (D3-GPC2-PBD) for 20 weeks, no tumor recurrence or weight changes were observed (37). In advanced NB models with larger tumors, D3-GPC2-PBD showed effective penetration and induced apoptosis. Similar results related to prolonged tumor regression, survival, and no recurrence were seen in PDX models with diverse NB-related mutations (MYCN, ALK, TP53) (37). The ADC’s bystander-killing effect was confirmed in co-cultures of responsive and resistant cell lines, showing cytotoxicity across all cells (37). The use of the same ADC in another study led to immunogenic cell death (ICD) both *in vitro* and *in vivo* by decreasing NB cell viability and increasing biomarker expression (38). Mice inoculated with NB cells pretreated with D3-GPC2-PBD exhibited slower tumor growth and better survival after tumor rechallenge with naïve NB cells, linked to T cell activation, a proinflammatory tumor microenvironment, enhanced immune cell infiltration, and increased phagocytosis (38). D3-GPC2-PBD also boosted CD40 expression on antigen-presenting cells (APCs). Combining the ADC with CD40 agonists or CD47 blockade further reduced tumor burden, improved survival, enhanced T cell infiltration, and induced long-term immunological memory, protecting against rechallenge (38). This highlights ADCs’ dual cytotoxic and immunoregulatory effects, as well as their potentiating effect when combined with immunotherapy.

**Table 1:** Antibody conjugates-based strategies in the treatment and diagnosis of neuroblastoma.

Antibody-drug conjugates	*Target, expression and context	Model	Outcome	Reference
*EV20/Omomyc*	***Target:*** HER3 ***Expression:*** transplanted MYCN (Kelly) amplified NB cell line with a lentiviral vector encoding HER ***In vivo:*** mice were injected intravenously with EV20/Omomyc (5 mg/Kg) No dose-response data	Pseudo-metastatic mouse model of NB (based on intravenous injections of Kelly cells)	Reduced growth of Kelly-HER3 metastatic tumors in the liver of mice treated with EV20/Omomyc	(22)
*CDX-0125-thienoindole*	***Target:*** ALK ***Expression:*** wild-type and mutated ALK-expressing NBs; high-level *ALK* amplification and protein expression ***In vitro:*** IC50 was 5.8 pM against NB-1 (ALK-amplified), 4.7 pM against IMR-32 (ALK wild type), 21.7 pM against SK-N-SH (ALK F1174L), and >1000 pM against SK-N-AS (ALK null) ***In vivo:*** dose-response assessment of 1, 3, and 10 mg/kg in Felix-PDX	NB-1 cell line; Patient-derived xenografted mice Felix-PDX females	Efficient antigen binding and internalization, cytotoxicity; Significant tumor growth delay	(25)
*m276-SL-PBD*	***Target:*** B7-H3 ***Expression:*** highly expressed in pediatric solid tumors ***In vivo:*** a single dose of 0.5 mg/kg intraperitoneally weekly administered (days 1, 8, and 15)	Several lines of NB cells and cell line-derived xenograft models; NB xenografted mice CB17SC *scid* ^−/−^ female	Decrease in the minimum relative tumor volume (RTV); prolonged event-free survival in all tested mice across all models	(28)
*B7-H3 Vobramitamab duocarmazine (vobra duo)*	***Target:*** B7-H3 ***Expression:*** all human NB cell lines tested expressed B7-H3; Not expressed in the murine NB cell lines, NX-S2 and Neuro2a ***In vitro:*** NB cells and multicellular tumor spheroids models were treated with escalating concentrations (40-80-160-320-640 ng/mL) ***In vivo:*** 1 mg/kg vobra duo administered intravenously one time a week for 3 weeks (QWx3); in the resected mouse NB models the regimen was QWx4	Panel of human NB cell lines and murine NB cell lines; Pseudo metastatic, and resected mouse NB models	Induced apoptosis; Delayed the tumor growth, and increased the survival rate	(31)
*8F2-SG3199* *and* *R20-SG3199*	***Target:*** LGR5 ***Expression:*** NB cell lines with a high expression of LGR5 ***In vitro:*** evaluation of the cell viability in SKNAS, SKNBE2, and CHP212 cell lines (IC50 of 8F2-SG3199 ranging from 0.1 and 0.2 nM) ***In vitro:*** R20-SG3199 has been demonstrated to be ~100× less potent ***In vivo:*** 8F2-SG3199 (0.1 mg/kg and 0.3 mg/kg), R20-SG3199 (0.3 mg/kg), once per week for a total of five doses	NB cell lines (SKNAS, SKNBE2, and CHP212); Xenograft models of SKNAS female nu/nu mice	Potent cytotoxicity: Inhibition of tumor growth but no cell eradication, increase in the survival rate	(33, 91)
*D3-GPC2-PBD*	***Target:*** GPC2 ***Expression:*** NB PDXs and cell line xenografts with a range of GPC2 cell-surface expression: (GPC2^Hi^ and GPC2^UltraHi^ cell populations) ***In vitro:*** SK-N-SH/NBL-S^NB^ cells showed higher levels of GPC2 expression which correlated with an acquired susceptibility to the D3-GPC2-PBD ADC (IC50 = 1.81 ± 1.33 pM) ***In vivo:*** evaluation of the efficacy of the ADC in NB PDXs and cell line xenografts with a range of GPC2 cell-surface expression and genomic aberrations	Several NB cell lines, NB-PDXs, and xenografts 1)GPC2^Mod^, *MYCN* non-amplified, *ALK* wild type, *TP53* wild type; 2)Engineered mouse NB 9464D-GPC2 isogenic cell line in the immunocompetent C57BL/6J mouse strain; 3)NB-PDX model COG-N-421x (GPC2^Hi^, *MYCN* amplified, *ALK* wild type, *TP53* wild type; Significantly locally advanced COG-N-421x PDXs; High-GPC2-expressing NB cell-line-derived xenograft SK-N-AS (GPC2^Hi^, *MYCN* non-amplified, *ALK* wild type, *TP53* mutated	High specificity of the D3-GPC2 binder towards a conformational epitope of both human and mouse; 1)Potent tumor regression and a significant dose-dependent prolonged survival, with no observed toxicities; 2)No tumor recurrence, after 20 weeks post-ADC dosing in mice; 3)Potent tumor regression and a significant dose-dependent prolonged survival, with no observed toxicities; Significantly prolonged tumor regression and survival, upregulation of intra-tumoral DNA damage and apoptosis, and no observed toxicity	(37)
*D3-GPC2-PBD*	***Target:*** GPC2 ***Expression:*** GPC2 stably overexpressed in NXS2-GPC2 and 9464D-GPC2 by flow cytometry. Overexpression was maintained after NXS2-GPC2 and 9464D-GPC2 *in vivo* tumor engraftment in immunocompetent syngeneic A/J and C57BL/6 mice ***In vitro:*** cell viability assessment in GPC2-transduced murine NB cells ***In vivo***: vaccinated subcutaneously with 2×10^6^ 9464D-GPC2 cells treated with ADC (30 ng/mL for 96 hours), rechallenged subcutaneously in the contralateral flank 7 days layer with 2×10^6^ naïve 9464D-GPC2 cells	Human NB cell lines (NB-EbC1, SMS-SAN, NB-SD, NB69); Murine NB cells (Murine NB cells 9464D and NXS2); 6-weeks-old C57BL/6 or A/J female mice	NB cell viability decreased, increased phagocytosis in NB-EbC1 cells, and increased immunogenic cell death; Decreased tumor growth in 72% of mice and increased survival rate after the vaccination of A/J mice with NXS2-GPC2 cells treated with ADC	(38)
*Anti-GD2 scFv and minibodies conjugates*	***Target:*** GD2 ***Expression:*** absent in NGP-127, highly expressed in IMR-32 ***In vitro:*** NGP-127 cell line was insensitive; IMR-32 (IC50 of scFv-MMAE and minibody-MMAE 81.1 ± 5 nM and 56.5 ± 4 nM, respectively), IC50 of scFv-MMAF and minibody-MMAF (116.7 ± 8 nM and 98.3 ± 6 nM, respectively)	GD2-negative NGP-127 and GD2-positive IMR-32	Cytotoxic effects (cell cycle arrest and subsequent cell death)	(50)
*Anti-GD2 conjugated to monomethyl auristatin E (MMAE)*	***Target:*** GD2 ***Expression:*** highly expressed in IMR-32, low expressed in SH-SY5Y, and absent in NGP-127 ***In vitro:*** Ch14.18-MMAE in IMR-32, (IC50 below 1 nM) in SH-SY5Y, lower in GD2-negative NGP-127 minimal (not reaching IC20); Ch14.18-MMAF was less potent in human cells (IC50 less than 2 nM in IMR-32)	IMR-32, SH-SY5Y, NGP-127 NB cell lines	Cytotoxic effects were strongly correlated with GD2 expression (cell cycle arrest and subsequent cell death)	(54)
*Rova-T*	***Target:*** DLL3 ***Expression:*** highly expressed in NB solid tumors, widely expressed in NB PDX models ***In vivo:*** dose-response study	Four NB PDX models: CB17 SCID mice bearing COG-N-452x, Felix PDX, COG-N-519x, COG-N-415x	Clear dose-response effect in all models, anti-tumoral activity and growth inhibition were highlighted at a single dose of 0.6 mg/kg of Rova-T. Doses of 0.3 and 0.6 mg/kg induced significant increases in event-free survival, except for the COG-N-415x dosed at 0.3 mg/kg. A single 1 mg/kg dose to 1 mg/kg weekly ×3 in mice bearing the COG-N-415x PDX, was sufficient to maintain a complete response for 7 weeks with the weekly ×3 treatment schedule	(52)
*1959-sss/DM3*	***Target:*** LGALS3BP ***Expression:*** expressed in NB cell lines, independently of MYCN amplification ***In vitro:*** treatment ranges from 0 to 10 nM ***In vivo:*** 1)control vector-infected (shCTR) and LGALS3BP-knockdown (shLGALS3BP) cells were implanted subcutaneously into nude mice. After tumor growth, animals were treated by injections of 1959-sss/DM3 twice a week for two weeks at 10 mg/kg; Orthotopical implant of SH-SY5Y in the adrenal glands of nude mice, then were treated with the 1959-sss/DM3 (10 mg/kg) 2)stage IV MYCN amplified PDXs, COG-N-636, COG-N-603 and COG-N-453, were used. Animals received an injection with 1959-sss/DM3 twice a week for two consecutive weeks at the dose of 10 mg/kg 3) 1959-sss/DM3 (10 mg/kg), was intravenously injected every three days for a total of 3 doses	Panel of human NB cell lines 1)Subcutaneous NB xenograft model and orthotopic SH-SY5Y-LUC adrenal xenografts; 2)NB stage IV amplified xenografts (PDXs, COG-N-636, COG-N-603 and COG-N-453); 3)Metastatic models: SKNAS (LGALS3BP high), Kelly (LGALS3BP low), and hNB (LGALS3BP negative)	Cell death; 1)Complete remission of tumors, prolongated survival in treated mice up to 80 days from the start of ADC injections; 2)Complete eradication of tumors in the COG-N-636 PDX model; and 5 out of 6 mice injected with COG-N-603 PDX cells were tumour-free; 3)60-70% inhibition of metastasis formation in the liver and abolishment of metastasis formation in the bone marrow	(56)
Immunotoxins	Target, expression and context	Model	Outcome	Reference
*IT BW-2* *(mouse anti-human-HuD monoclonal antibody + streptavidin/saporin complexes)*	***Target:*** HuD ***Expression:*** strongly expressed in NB cell line Neuro-2a (~39-40 kD) ***In vitro:*** dose-response treatment ranging from 0 to 5 μg/ml ***In vivo:*** HuD-positive Neuro-2a NB in immunocompetent A/J mice were intratumorally injected with two weekly doses of BW-2 1 mg/kg	Mouse NB Neuro-2a line; Neuro-2a allografted A/J mice	Selective cytotoxicity against HuD-positive NB cells; Inhibition of tumor growth and metastasis in 80% of treated immunocompetent A/J mice	(60)
*BW704dgA* *(BW704 antibodies conjugated to deglycosylated ricin A)*	***Target:*** GD2 ***Expression:*** selectively expressed in the surface of NB cells ***In vitro:*** IMR5 cells were incubated for 24 to 96 hours with a different concentration of BW704dgA, IC50 values of: 3 × 10^−9^ M (24 hr), 8 × 10^−10^ M (48 hr), 5 × 10^−10 M^ (72 hr), and 1 × 10^−11^ M (96 hr) ***In vivo:*** mice inoculated with the human NB cell line IMR5a were treated with the ITs (48 mg/mouse); the treatment consists of a single dose on day 4, or divided into three doses: days 4, 5, and 6 after the injection	NB IMR5 cells; SCID mice NB xenografts	Inhibit protein synthesis in GD2-positive NB cells; Improved survival outcomes in SCID mice bearing human NB xenografts	(61)
*Diphtheria toxin A chain combined with the 5F11 scFv; 14.18 scFv with Pseudomonas exotoxin.*	***Target:*** GD2 ***Expression:*** abundantly expressed in the surface of NB cells ***In vitro:*** IMR5 cells were treated with different concentrations of the IT (IC50 = 0.326 μg/ml)	NB IMR5 cell line	Inhibition of cell viability, *in vitro* cytotoxicity against GD2-positive tumor cells	(62)
*LH7-PE38* *(Anti GPC2 + Pseudomonas exotoxin PE38)*	***Target:*** GPC2 ***Expression:*** highly expressed the NB cell lines LAN1, IMR5, LAN5, IMR32, and NBEB (with MYCN overexpression), low expression was found in the SK-N-SH cell line ***In vitro:*** assessment of the growth of GPC2^+^ cell (LAN1 and IMR5 IC50 values ranging between 0.5-1.2 nM) ***In vivo:*** 0.4, 0.6, 0.8 mg/kg were administered for 10 days to the mice, the dose of 0.4 mg/kg was well tolerated	IMR5, LAN1, IMR32, and LAN5 NB cell lines; Athymic nud/nud mice	Potent and selective inhibition of the growth of GPC2^+^ cell lines LAN1 and IMR5; Inhibition of the tumor growth without any effect on the body weight, increase in white blood cells in those mice	(63)
*Burkholderia Lethal Factor 1 (BLF1)*	***Target:*** eIF4A ***Expression:*** overexpressed in NB patients (90) ***In vitro:*** dose-response assessment of BLF1 toxicity	MYCN-amplified IMR-32, SK-N-BE(2)) and non-MYCN-amplified SH-SY5Y and LA-N-6 NB cell lines	Selective cytotoxicity, caspase 3/7 activation and apoptosis induction in MYCN-amplified cells with 300 nM causing the maximal growth inhibition	(64)
*scFv(14E1)-Pseudomonas exotoxin A (ETA) and TGF-α-ETA*	***Target:*** EGFR ***Expression:*** expressed in all the cell lines, increased expression in cisplatin-resistant sublines ***In vitro:*** the cytotoxic activity was assessed by incubating cells for 5 days with increasing concentrations of the recombinant toxins; the induction of apoptosis was investigated by incubating the UKF-NB-3 and UKF-NB-3^r^CDDP^1000^ cell lines for 72 hours with 100 ng/mL of scFv(14E1)-ETA or TGF-α-ETA	NB cell lines (IMR-32, NLF, UKF-NB-3, SH-SY5Y)	Anticancer effects against NB in cell lines insensitive to cetuximab or EGFR tyrosine kinase inhibitors	(65)
*Saporin-conjugated antibodies (p75IgG-Sap)*	***Target:*** p75NTR ***Expression:*** upregulated in NB HDACi pre-treated cells (24 h of entinostat (0.3 and 1.0 µM) plus p75IgG-Sap (30 nM)) for an additional 24 h were able to upregulate the expression of p75NTR ***In vitro:*** 1)cytotoxic activity of p75IgG-Sap after the entinostat treatment was assessed preincubating cells for an additional 24 h with 1 µM entinostat and then exposed for additional 24 h to increasing concentrations of p75IgG-Sap; 2)multicell spheroids were treated with 1 µM entinostat and then incubated with p75IgG-Sap (30 nM). ***In vivo:*** animals were pre-treated for 10 days with entinostat and then injected with p75IgG-Sap (5.0 µg)	MYCN not amplified SH-SY5Y and MYCN amplified LAN-1, Kelly, BE(2)C, IMR-32, and NB-1); NB multicell spheroids; Athymic nude BALB/c mice	1)Marked decrease in the cell surface expression of p75NTR in SH-SY5Y cells. Apoptosis in NB cells pre-treated with HDACi; 2)The combination of entinostat and p75IgG-Sap determined a significant tumor growth inhibition in entinostat-pre-treated spheroids; Significant decrease in tumor growth in entinostat + p75IgG-Sap-treated animals	(66)
Antibody-fluorophore conjugates	Target, expression and context	Model	Outcome	Reference
*Anti-GD2-IRDye800CW*	***Target:*** GD2 ***Expression:*** consistent expression of GD2 in HR-NB patients (differently expressed in patient-derived NB organoid: high in TIC772, intermediate in NB67 and low in NB39 respectively) ***In vivo:*** dose-escalating study was performed in in subcutaneous KCNR-derived tumor; In the orthotopic model 1 nmol anti-GD2-IRDye800CW was intravenously injected and the tumors were resected 4 days post-injection; In patient-derived NB organoid Fluorescence-guided surgery (FGS)	Patient-derived SMS-KCNR (KCNR) cell line; Orthotopic model with KCNR cells transplanted in the adrenal gland; Patient-derived NB organoid lines; TIC772, NB67 and NB39	1 nmol was the optimal dose and efficient real-time visualization of NB after 4 days; Identifying tumors without interference has proven valuable for tumor visualization and resection; GD2-IRDye800CW was sufficient for FGS also in patient-derived NB organoids which expressed low levels of GD2	(81)
*Dinutuximab IRDye800*	***Target:*** GD2 ***In vivo:*** mice with subcutaneous NB xenografts were injected with Dinutuximab-IRDye800, and images were taken at 24, 48, 72, and 96 h to assess the fluorescent signal	NB subcutaneous mouse xenograft	Achieved 97.5% per-pixel accuracy in distinguishing tumor from non-tumor tissues in preclinical models	(82)
*Dinutuximab IRDye800 (anti-GD2-IR800 and anti-GD2-IR12)*	***Target:*** GD2 ***Expression:*** cell lines engineered to express GD2 ***In vitro:*** flow cytometry analysis, labeling of the adherent cell lines and LAN-1 spheroids ***In vivo:*** mice were injected intravenously (100 μg of anti-GD2-IR800 or anti-GD2-IR12) images were acquired using a clinical-grade **NIR-I imaging device** (EleVision IR Platform) 96 hours after the injection; Xenografted subcutaneous LAN-1 NB mice were injected with both conjugates and the images were acquired at 24, 48, 72, and 96 hours after the injection using the **IVIS Spectrum imaging system**	GD2-positive LAN-1, KELLY cell lines; LAN-1 cells; Xenograft athymic nude female mice (CD1-Foxn1nu)	Specific binding signal especially in G2-positive cells from both anti-GD2-IR800 and anti-GD2-IR12; A strong fluorescent cell surface signal in LAN-1 cells; *In vivo* higher tumor-to-background ratio, stable detection of the tumor above background tissue at all time points for both conjugates	(83)
*Dual-labeled GD2-specific tracers* *111In-αGD2-IR800*	***Target:*** GD2 ***Expression:*** expressed in all the cell lines under investigation ***In vitro:*** evaluation of the tracer binding affinities (K_d_) for anti-GD2 and DTPA-aGD2-IR800 were 2.39 nM (0.99-4.62 nM, 95% CI) and 21.31 nM (14.06-32.16 nM, 95% CI) ***In vivo:*** injection of the SK-N-BE(2) human-derived NB in the left adrenal gland of the mice; tracer detection 3 and 4 days later a tail vein injections of ^111^In-αGD2-IR800 (4.8 MBq/50 µg in 100 µl DPBS) in mice bearing NB xenograft	NMB6, SK-N-BE(2), SK-N-AS, SK-N-SH, SH-SY5Y and CHP212 NB cell lines; Orthotopic NB xenograft (SK-N-BE(2) mice surgically injected into the left adrenal gland)	Greater tumor accumulation, precise tumor visualization: high tumor-to-background, tumor-to-blood, and tumor-to-muscle ratios	(84)
Antibody-radioactive isotopes conjugates	Target, expression and context	Model	Outcome	Reference
131I-3F8 (Omburtamab)	***Target:*** B7H3 or GD2 ***Expression:*** patients with tumors expressing B7H3 or GD2 ***In vivo study 1***: intrathecal injection of ^131^I-3F8 (10 mCi) every 2-3 weeks (four injections, total dose 40 mCi), or 1 or 2 monthly injections of ^131^I-8H9 (10-60 mCi/injection) ***In vivo study 2:*** dose escalation study from 370 to 2960 MBq toxicity assessment	1) Humans; 2) Humans: phase I trial NCT00089245	1)Current doses were associated with acute side effects including transient headache, nausea, fever, and vomiting; complexively well tolerated by young patients; 2)Modest related adverse events; Increased survival rate (7,5 years) in patients receiving cRIT for NB	(74, 75)
Ch14.18/CHO labeled with the radiotracer 64 Cu	***Target:*** GD2 ***Expression:*** expressed in the cell lines under investigation ***In vitro:*** characterization of ^64^Cu-labeled ch14.18/CHO ***In vivo (preclinical):*** assessment of the biodistribution (PET); Injection of 50 µg radiolabeled ch14.18/CHO in the xenografted mice; ***In vivo (clinical):*** first translational approach involving ^64^Cu]Cu-NOTA-ch14.18/CHO in an individual patient suffering from NB	NB cell lines LS and CHP-134; Xenografts Six-week-old CD1 nude female mice; Humans	The radioimmunoconjugate strongly binds the GD-expressing NB cell lines LS and CHP-134 NB; Higher NB uptake of ch14.18/CHO; High signal intensity showed a specific GD2 membrane staining; Identification of NB metastases, although 2,5 weeks after the last treatment there was a reduction in the uptake of the radioimmunoconjugate in the femoral lesion	(76)

*the target expressions refer to the individual *in vitro* or *in vivo* studies in which the conjugate compounds are used.

#### 2.1.2 Optimizing ADC for NB: tailoring the treatment to the tumor

The beneficial use of ADCs as a therapeutic option for NB is in part summarized in its specific drug delivery that singles out tumor cells expressing the targeted antigen, circumventing problems such as unwanted side effects and treatment resistance that are observed with conventional therapy (39). Taking into account that NB is a pediatric cancer with a median age of diagnosis of 17 months (1, 40), prolonged treatments, drug resistance, off-target effects, and any subsequent organ damage can lead to short- or long-term health and growth complications in children (41, 42), making ADCs an optimal therapeutic choice. Nevertheless, treating solid tumors with ADCs is more challenging. The high intertumoral variability and NB’s cellular heterogeneity, particularly in target expression levels, can represent an obstacle in ADC-oriented therapies (43, 44). Dense vascularization, a complex tumor microenvironment, and the large molecular weight of ADC antibodies further limits penetration and efficacy (45, 46). To address these issues, miniaturized antibodies have been developed to improve ADC diffusion and effectiveness in solid tumors, including NB (15). Ganglioside (GD2) is a sialic acid-containing glycosphingolipid that can be used as a biomarker for NB as it is highly expressed on NB cell surface, especially in HR-NB (47). Anti-GD2mAbs such as dinutuximab and naxitamab, have been extensively used as part of therapeutic protocols for the treatment of patients with HR-NB (48, 49). However, to overcome the limitations linked to the antibody’s size, two antibody fragment formats were developed: a single-chain variable fragment (scFv) from the ch14.18 antibody and a minibody combining scFv with an IgG1 heavy chain (50). These were conjugated to tubulin inhibitors, forming fragment-drug conjugates (FDCs). In GD2-positive IMR32 NB cells, FDCs showed cytotoxicity, with minibodies outperforming scFv due to superior kinetics and bivalent binding, enabling efficient payload delivery, cell cycle arrest, and cell death (50) (**Table 1**). Intertumoral differences in antigen expression also play a crucial role in determining the efficacy of ADCs in NB. A study exploring the neuroendocrine characteristics of NB targeted the delta-like ligand 3 (DLL3), an inhibitory Notch ligand selectively upregulated in neuroendocrine tumors where it potentiates tumor proliferation and invasion by activating the Notch and the SNAI1/Snail signaling pathways (51–53). The study demonstrated that Rova-T, an anti-DLL3 ADC previously proven to be ineffective in small cell lung cancer (SCLC), exhibited preclinical therapeutic potency in NB-PDX models. Nonetheless, this efficacy was closely dependent on DLL3 expression, emphasizing the need to restrict its application to NB cases with high DLL3 levels. This underscores the critical impact of antigen expression variability across tumors in dictating responsiveness to ADCs (52) (**Table 1**).

The impact of NB intertumoral heterogeneity and ADC composition on therapy has been studied extensively (44). Anti-GPC2 ADCs with different payloads showed enhanced internalization in NB cells with dense GPC2 expression. However, TP53-mutated NB cells exhibited resistance to ADCs with DNA binding and DNA topoisomerase 1 inhibiting payload. MYCN-amplified cells were more sensitive to ADCs with N-acetyl-calicheamicin g1 compared to non-MYCN-amplified cells, while cells with high ATP-binding cassette transporter B1 (ABCB1) expression, driven by MYCN, resisted tubulin inhibitor payloads. Notably, ALK-related mutations did not influence resistance to ADCs, regardless of payload type (44). The link between payload selection and NB heterogeneity was studied using monomethyl auristatin E (MMAE) and F (MMAF) as payloads (54). The efficacy of this ADC was evaluated across various NB cell lines with varying GD2 expressions, showing cytotoxicity positively correlated with GD2 levels (54). However, payload type also influenced outcomes; anti-GD2 conjugated with MMAE was more cytotoxic in SH-SY5Y cells than IMR-32 cells (54) (**Table 1**). These findings underscore the critical roles of NB heterogeneity, antigen density, and payload choice in ADC therapeutic efficacy. The tumor microenvironment plays a critical role in ADC efficacy as well, particularly through components like extracellular vesicles (EVs), which contribute to NB progression by transferring DNA, RNA, and proteins from tumor to healthy cells, promoting metastasis (55). Leveraging its high expression on EVs, Galectin-3 binding protein (LGALS3BP) was targeted by 1959-sss/DM3, a non-internalizing ADC shown to bind EVs effectively and yield therapeutic benefits in NB models. This ADC demonstrated efficacy in SKNAS subcutaneous NB xenografts, orthotopic SH-SY5Y-LUC adrenal xenografts, and patient-derived stage 4 NB xenografts (COG-N-636 and COG-N-603) with high LGALS3BP expression. Notably, tumor elimination was achieved in both PDX models, with COG-N-636 exhibiting a longer recurrence-free period than COG-N-603 (56) (**Table 1**). Success with targeted therapies depends on the application context. For heterogeneous tumors like NB, ADCs must match the tumor’s molecular profile and microenvironment for optimal efficacy. Molecular profiling aids ADC design while targeting EVs and other non-tumor-cell elements offer new ways to combat NB progression. Key design factors-antigen, mAb size, linker, and payload-are crucial for ADC efficacy and clinical potential.

### 2.2 Immunotoxins’ role in NB therapy

ADCs and ITs are therapeutic complexes created by binding a toxic element to a mAb. However, in ADCs, the mAb is conjugated to a payload consisting of a cytotoxic drug. This includes as mentioned before: tubulin inhibitors, DNA-damaging agents, or immune-stimulatory compounds (15, 57). In contrast, the conjugates in ITs are not drugs, but rather toxins, which are poisons that are bacterial, animal, or plant-based (11, 58). One promising therapeutic target is the HuD antigen, a neuronal-specific RNA binding protein that is highly expressed in both NB and SCLC cells and was shown to promote NB tumor growth and survival through suppressing apoptotic pathways and downregulating mTORC1 activity (59). The conjugation of an anti-HuD antibody with the ribosomal toxin saporin, forming the IT-BW-2, has shown selective cytotoxicity against HuD-positive NB cells (50). Another well-established target is GD2, overexpressed in NB cells. Antibodies like BW704, conjugated to deglycosylated ricin A (BW704dgA), inhibit protein synthesis in GD2-positive NB cells. *In vitro* studies demonstrated the potent cytotoxic effects of BW-2 at low concentrations, while BW704dgA improved survival outcomes in SCID mice bearing human NB xenografts (60, 61). ITs based on the anti-GD2 scFv 5F11 have also demonstrated high potency. This recombinant fusion protein combines the diphtheria toxin A chain with the 5F11 scFv, allowing it to directly kill GD2-positive tumor cells without relying on Fc-mediated mechanisms like ADCC or CMC. Similarly, a recombinant fusion of 14.18 scFv with Pseudomonas exotoxin A has shown elevated *in vitro* cytotoxicity against GD2-positive tumor cells (62). GPC2 has also been targeted by ITs. This was done by using single-domain antibodies that bind specifically to GPC2 and conjugating them with a truncated Pseudomonas exotoxin (PE38) to create chimeric proteins capable of delivering the toxin directly to NB cells. These ITs exhibited potent cytotoxicity in GPC2-positive NB cell lines at low concentrations (IC50 values of 0.5-1.2 nM) while sparing GPC2-low-expressing cells. In mouse models, the immunotoxin LH7-PE38 significantly inhibited tumor growth at 0.4 mg/kg with minimal side effects, though higher doses showed toxicity, underscoring the importance of dose optimization (63). Another promising therapeutic agent is Burkholderia Lethal Factor 1 (BLF1), which exhibits selective cytotoxicity against MYCN-amplified NB cells. By inhibiting eukaryotic initiation translation factor 4A (eIF4A), BLF1 suppresses the translation of oncogenic proteins, inducing apoptosis in cancer cells while sparing non-transformed cells. BLF1 demonstrated a 70% reduction in viability in MYCN-amplified cells, with minimal impact on non-MYCN-amplified cells. Furthermore, BLF1 downregulated critical proteins such as MYCN and CDK4 and increased caspase 3/7 activity threefold, highlighting its potential for targeted NB therapy with reduced side effects compared to conventional treatments (64). NB cells often express the epidermal growth factor receptor (EGFR), making them susceptible to EGFR-targeted therapies. Cisplatin-resistant NB cells express higher levels of EGFR, making them particularly susceptible to EGFR-targeted toxins such as scFv(14E1)-Pseudomonas exotoxin A (ETA) and TGF-α-ETA. These toxins, especially when combined with cisplatin, significantly enhanced apoptosis and reduced cell viability compared to monotherapies. This combination approach shows promise for addressing chemoresistant NB (65). Novel strategies that aim at rendering NB more vulnerable are garnering interest. Notably, combination therapy is becoming increasingly popular. Given that the success of ACs relies on targeting molecules that are over- or uniquely expressed on tumor cells, identifying novel targets of this nature is crucial for advancing the development of therapeutical options, especially ACs. Fittingly, previous studies have shown that NB cells are particularly ‘impressionable’ when it comes to cellular target overexpression. For instance, exposure of NB cells to entinostat, a histone deacetylase inhibitor (HDACi), has led to the upregulation of the neurotrophin receptor p75NTR (66). Targeting p75NTR with saporin-conjugated antibodies (p75IgG-Sap) effectively induced apoptosis in these pretreated cells, taking advantage of the heightened receptor expression (66) (**Table 1**). Therefore, the priming of NB cells with agents that can induce the overexpression of specific receptors can be strategically used to increase NB susceptibility to AC-based approaches. This synergistic effect can be further explored using other compounds, especially those used in conventional cancer therapy such as chemotherapeutic agents and cytokines, to explore their potential modulatory effect on the expression of pre-existing underutilized targets or the induction of novel ones that can then be identified and precisely targeted. In summary, ITs are a promising strategy for treating NB, offering precise tumor targeting while sparing normal tissues. Advances in delivery systems, enhanced antigen expression and mitigation of side effects continue to refine their clinical utility, making them a promising avenue for improving NB treatment outcomes.

### 2.3 Radioimmunotherapy in NB

Radioimmunotherapy (RIT) involves using mAbs conjugated to radioactive isotopes, enabling targeted delivery of radiation to tumor cells expressing specific antigens. This approach aims to maximize the eradication of tumors while minimizing damage to surrounding healthy tissues. Similarly to the other ACs, identifying suitable target antigens, that are highly expressed in NB cells but show limited expression in normal tissues is a critical component of RIT. With GD2 being a notable target for ACs as previously described, the anti-GD2 mAb, such as 3F8 facilitates the direct delivery of therapeutic agents to NB cells. It is important to emphasize that these drugs may act as radiosensitizers. When appropriately timed, radiolabeled mAbs can be most effective when tumor cells are radiosensitive during the cell cycle phases. This rationale underpins the combination of chemotherapy and RIT (67–72). Radiolabeled anti-GD2 antibodies, such as 131I-3F8 and 131I-14G2a, have demonstrated efficacy in targeting NB cells and have been utilized for radioimmunodetection. Phase I and II studies have evaluated the toxicity, biological distribution, and dosage of intravenously administered 131I-3F8 in NB patients, revealing side effects such as pain and allergic reactions. In cases of leptomeningeal NB, 131I-3F8 was delivered intraventricularly, with no long-term toxicities observed (73). Metastatic NB remains one of the most challenging forms of NB to treat. Notably, survival rates have improved for patients with recurrent NB treated with a rescue regimen incorporating RIT. This includes intrathecal delivery of 131I-labeled mAbs targeting GD2 or B7-H3 following surgery and radiation to address minimal residual disease. A study reported that 14 out of 21 patients treated with this RIT-based salvage regimen were alive 7 to 74 months after relapse, with the others remaining NB-free (74) (**Table 1**). Another study from the same group evaluated the intraventricular injection of the anti-B7-H3 murine mAb omburtamab. The findings indicated that omburtamab can be safely used for RIT, even in patients previously exposed to high doses of chemotherapy and radiation. Remarkably, 44% of patients with recurrent NB survived for 13–17 years following RIT, with only 13% experiencing another relapse (75) (**Table 1**). Preclinical studies have also emphasized the potential of the ch14.18/CHO antibody labeled with the radiotracer 64Cu for highly specific targeting of NB cells. Clinical PET/MR scans confirmed its ability to detect NB metastases and enhance GD2-directed immunotherapy. Importantly, tumor binding by the radiolabeled antibody was blocked by excess unlabeled antibodies, validating its specificity. This technique enables the detection of otherwise inaccessible lesions, improving treatment planning and predicting therapeutic efficacy (76) (**Table 1**). Despite these advancements, the integration of RIT in complex treatment protocols remains unexplored, with no clinical or preclinical studies combining these modalities. This reflects the experimental nature of these approaches and the complexity of developing a unified treatment regimen. Nevertheless, RIT holds significant promise for the treatment of NB, offering a targeted approach with reduced systemic toxicity. Continued research and clinical evaluation are essential to fully realize the potential of this therapeutic modality in pediatric oncology.

### 2.4 Antibody-fluorophore conjugates in NB

AFCs combine antibody specificity with fluorescence for biomolecule visualization and quantification. Introduced by Albert Coons in 1941, they transformed immunology and microscopy (77). AFCs are essential in imaging, diagnostics, and cancer research, aiding in detecting breast, ovarian, colorectal, and lung cancer (78). Their precision in targeting tumor-associated antigens enables accurate imaging of malignant tissues (79). Fluorophores, from dyes to quantum dots and NIR fluorophores, improve sensitivity and tissue penetration (80). AFCs play key roles in immunohistochemistry, fluorescence-guided surgery, and flow cytometry, with real-time imaging advancements enhancing therapies, including for NB.

#### 2.4.1 Overview of AFC use in NB: where are we now?

Advancements in imaging techniques and probes are transforming NB surgery, offering promising strategies to improve tumor detection, resection precision, and outcomes for pediatric patients. One notable innovation is the anti-GD2-IRDye800CW fluorescent probe, which specifically binds to NB cells, enhancing fluorescence-guided surgery (FGS). Preclinical studies using orthotopic tumor models demonstrated its effectiveness in identifying tumors without interference from prior immunotherapy with dinutuximab-beta, a clinically approved antibody. This combination approach shows potential for pediatric clinical trials, supported by GD2 expression across NB stages and patient-derived organoid models. Anti-GD2-IRDye800CW has proven valuable for tumor visualization and resection, with the potential to improve survival rates for children with high-risk NB (81) (**Table 1**). Multispectral short-wave infrared (SWIR) fluorescence imaging is another promising development for FGS. Using NB-specific NIR-I probes like Dinutuximab IRDye800, SWIR imaging achieved 97.5% per-pixel accuracy in distinguishing tumor from non-tumor tissues in preclinical models. This precision was further enhanced through machine learning methods, including principal component analysis (PCA) combined with the k-nearest neighbor (KNN) algorithm, which classified tumor tissues with 97.1% accuracy and background areas with 99.2% accuracy (82). SWIR imaging outperforms NIR-I imaging, offering higher tumor-to-background ratios (4.6 vs. 2.6), detecting small tumor volumes (0.9 mm³), and penetrating up to 3 mm into tissues. These capabilities underscore its potential to improve surgical precision by minimizing residual malignancy (83). In addition, dual-labeled GD2-specific tracers, such as 111In-αGD2-IR800, have been evaluated for intraoperative molecular imaging (IMI). In NB xenograft models, these tracers demonstrated high tumor-to-background, tumor-to-blood, and tumor-to-muscle ratios, facilitating precise tumor visualization with imaging tools like the Neoprobe and SPY-PHI NIR camera. This enabled the identification of residual disease and complete tumor excision. Compared to control tracers, 111In-αGD2-IR800 exhibited significantly increased tumor accumulation and negligible uptake in non-target organs, highlighting its sensitivity and specificity for NB detection (84) (**Table 1**). Organoid-based platforms are also advancing personalized FGS probe development. These platforms identified tumor-specific membrane targets, such as GD2 and L1CAM, for NB and breast cancer (BC) using patient-derived organoids (PDO) and xenograft models. By analyzing RNA sequencing datasets and verifying limited expression in healthy tissues, the study identified five potential NB probes and underscored the need for additional BC probes to address tumor heterogeneity. These findings suggest that organoid-based platforms can improve FGS accuracy by reducing false positives and ensuring robust tumor labeling (85). Collectively, these imaging innovations-from fluorescent and radiolabeled probes to SWIR imaging and organoid-based platforms-represent a transformative leap forward in NB surgery. They enhance tumor visualization, guide precise resections, and enable tailored therapeutic approaches. By addressing current challenges in detecting and excising tumors, these technologies promise to reduce residual malignancies, improve surgical precision, and ultimately enhance outcomes for children with this challenging disease.

## 3 Conclusion

Advancements in ACs, including ADCs, ITs, RIT, and AFCs, have significantly improved the precision in the treatment and diagnosis of NB by delivering cytotoxic agents and labeling molecules directly to tumor cells. NB’s heterogeneous nature, its diverse molecular profile, and complex microenvironments require careful consideration when selecting or designing the appropriate ACs to maximize therapeutic efficacy and diagnostic reliability. As of now, no ADCs, RIT or ITs have been FDA-approved specifically for NB management. Finding the adequate conditions of ACs’ conception and application in NB are still investigated preclinically, as previously reported above. Nonetheless, some ACs are currently in clinical trials (NCT06041516; NCT06224855; NCT00089245 (**Table 1**) and NCT06395103). Although it is the most studied subclass of ACs, only one clinical study investigating ADCs has been completed. The latter reported limited efficacy of the ADC Lorvotuzumab mertansine, with 25% of the treated NB patients showing stable disease progression through 6 cycles of treatment (86). Partial to complete delayed responses were only observed, in a reserved manner, in the case of Rhabdomyosarcoma and synovial sarcoma (86). On the other hand, the reliance on ADCs that have previously demonstrated efficacy in other malignancies is a good ground to start upon. For instance, the clinical testing of Zilovertamab vedotin (NCT06395103) has a strong rationale thanks to its targeting of the receptor tyrosine kinase-like orphan receptor 1 (ROR1), a surface protein that is overexpressed in NB and that plays a crucial role in its oncogenesis, with high levels of its mRNA correlating with poor prognosis, as it activates several signaling pathways that promote survival, invasion and metastasis (87, 88). Its previous efficacy in treating lymphoid cancers makes it a potentially good candidate for the treatment of other malignancies (89). Nevertheless, Zilovertamab vedotin, as well as the efficacy of other ACs, remains to be proven in the case of NB, as several challenges related to NB’s nature and ACs delivery within the tumor’s microenvironment continue to persist. Further advancements in ACs design, combined with deeper insights into NB-specific molecular targets, offer hope for improved efficacy and higher transability into clinical settings. Recent research has also highlighted the potential of cytokine-based treatments and epigenetic drugs to sensitize NB cells and render them more vulnerable to these targeted therapies. However, no approved therapeutic strategies currently combine these antibody conjugates with standard chemotherapy for aggressive NB, emphasizing the need for further research to validate these innovations in combination therapy and clinical trials to improve outcomes for HR-NB patients by addressing CR and systemic toxicity.
